# The Noor Project: fair transformer transfer learning for autism spectrum disorder recognition from speech

**DOI:** 10.3389/fdgth.2025.1274675

**Published:** 2025-08-18

**Authors:** Najla D. Al Futaisi, Björn W. Schuller, Fabien Ringeval, Maja Pantic

**Affiliations:** ^1^ GLAM – Group on Language, Audio & Music, Imperial College London, London, United Kingdom; ^2^ Chair of Embedded Intelligence for Health Care and Wellbeing, University of Augsburg, Augsburg, Germany; ^3^ Grenoble INP, LIG, University Grenoble Alpes, Inria, CNRS, Grenoble, France; ^4^ iBUG – Intelligent Behaviour Understanding Group, Imperial College London, London, United Kingdom

**Keywords:** autism spectrum disorder, child speech, artificial intelligence, deep learning, transformer, transfer learning, fairness in AI

## Abstract

Early detection is crucial for managing incurable disorders, particularly autism spectrum disorder (ASD). Unfortunately, a considerable number of individuals with ASD receive a late diagnosis or remain undiagnosed. Speech holds a critical role in ASD, as a significant number of affected individuals experience speech impairments or remain non-verbal. To address this, we use speech analysis for automatic ASD recognition in children by classifying their speech as either autistic or typically developing. However, due to the lack of large labelled datasets, we leverage two smaller datasets to explore deep transfer learning methods. We investigate two fine-tuning approaches: (1) Discriminative Fine-Tuning (D-FT), which is pre-trained on a related dataset before being tuned on a similar task, and (2) Wav2Vec 2.0 Fine-Tuning (W2V2-FT), which leverages self-supervised speech representations pre-trained on a larger, unrelated dataset. We perform two distinct classification tasks: (a) a binary task to determine typicality, classifying speech as either that of a typically developing (TD) child or an atypically developing (AD) child; and (b) a four-class diagnosis task, which further classifies atypical cases into ASD, dysphasia (DYS), or pervasive developmental disorder-not otherwise specified (NOS), alongside TD. This research aims to improve early recognition strategies, particularly for individuals with ASD. The findings suggest that transfer learning methods can be a valuable tool for autism recognition from speech. For the typicality classification task (TD vs. AD), the D-FT model achieved the highest test UAR (94.8%), outperforming W2V2-FT (91.5%). In the diagnosis task (TD, ASD, DYS, NOS), D-FT also demonstrated superior performance (60.9% UAR) compared to W2V2-FT (54.3%). These results highlight the potential of transfer learning for speech-based ASD recognition and underscore the challenges of multi-class classification with limited labeled data.

## 1 Introduction

Early detection of developmental disorders bears greatest importance for a child’s life and future. It is only with early diagnosis that we are able to impose early intervention treatment plans. Some disorders such as autism spectrum disorder (ASD) are incurable, however, they can be managed with treatments such as special care and education as well as focus on social development. ASD is a neurological disease correlated by imbalances in the brain and could cause later difficulties such as social, communication and learning issues. Speech comes as an important factor amongst these issues, more so as individuals with ASD experience speech difficulties that could be in the form of a lack in verbal skills or continuing to be non-verbal/speaking.

Even verbal autistic children exhibit discernible acoustic patterns in their speech (1, 2). For instance, a monotonous speech pattern is evident in ASD speech and is considered as one of the characteristic features of ASD communication that children with ASD continue to exhibit even as they progress into school age (3). In other words, their speech lacks the typical variations in pitch, intonation, and expressive elements that are commonly observed in the speech of typically developing (TD) children (without ASD). Generally, autistic traits can be found in ASD speech patterns, which can be recognised by a neural network (4). Consequently, ASD can be recognised through the analysis of speech. The abilities of deep learning models enable the automatic recognition of medical conditions based on speech (5). machine learning (ML) facilitates autism classification by means of speech, including speech transcripts (6) and through acoustic features characterisation of autism speech (7). Speech-related research comprises of extracting and analysing various acoustic features from speech recordings of individuals with ASD, aiming to identify unique patterns or markers that distinguish them from TD individuals. One widely used feature extraction set is the ComParE feature set, which stands for the Computational Paralinguistics Challenge (8), serving the purpose of capturing diverse aspects of speech signals by generating a comprehensive set of acoustic features. These features encompass prosodic, spectral, and voice quality attributes, among others. ML models are trained on these feature sets to distinguish between ASD and non-ASD speech samples.

Machine Learning algorithms and audio data are leveraged to classify speech samples into ASD and non-ASD (TD) categories. These ML techniques include support vector machines (SVM) (9) and Random Forests (10), and deep learning models like convolutional neural networks (CNNs) (11) and recurrent neural networks (RNNs). While other research explores multi-modal classification, by combining audio features with other modalities such as textual or visual information for an improved classification performance. ML models have been used to recognise autism in other varying use-cases, including through the use of biomedical images of the brain (12) and facial features and biomedical images leveraged in unison (13).

Obtaining datasets with human subjects and especially child data is a difficulty often faced by researchers. Research ethics committees (RECs) or ethics review boards (ERBs) such as institutional review boards (IRBs) work to safe guard the information of human subjects. With that in mind, we investigate deep transfer learning that assists models that have inadequate/insufficient training data, as it alleviates the assumption that the training data needs to be independent and identically distributed with the test data (14). We focus on transfer learning techniques through fine-tuning models, a state-of-the-art approach, and an existing method on two datasets containing vocalisations of children aged 4 to 18 years old. We implement a discriminative fine-tuning model that pre-trains on child data and then fine-tunes on a fairly small dataset with child speech data. We then implement a Wav2Vec 2.0 transformer framework that leverages a model pre-trained on a 960 h of unlabelled speech data, to be fine-tuned to the small child dataset. We therefore test the abilities of fine-tuning methods both tuned using the same dataset, though pre-trained on unrelated and related tasks with differing sizes and tasks.

Although Wav2Vec was initially created and utilised for speech-to-text encoding, i. e., automatic speech recognition (ASR) (15, 16), it has been successfully applied to emotion prediction (17) and even language identification (18). In this research, we implement the updated version, being Wav2Vec 2.0 (19), for the classification of speech of ASD and typically developing children. This classification can be accomplished by analysing suited acoustic features (20). We compare our results to those of other studies employing versions of this transformer model. As for discriminative fine-tuning, it has successful applications in speech recognition (21) and natural language understanding (22); to the best of our knowledge, however, this is its first implementation to ASD recognition from speech. In addition to the binary typicality classification task (TD vs. AD, where AD encompasses all non-typically developing cases), we implement a four-class diagnosis task that classifies speech into one of four categories: TD, ASD, DYS, and NOS. The experiments carried out for this research are detailed throughout this paper to facilitate reproducibility by interested readers. We conduct further experiments in relation to fairness testing, to test whether the models have bias towards one gender over the other.

## 2 Datasets

### 2.1 De-Enigma corpus

The De-Enigma corpus has been created for the De-Engima research project, which has the goal of improving education for children diagnosed with ASD (23). This is a Horizon 2020 initiative, see https://de-enigma.eu/ for more information on the project. The participating 55 children are between the ages of 4 and 10 years (25 from Serbia and 25 from the United Kingdom). The corpus does not include a control group and all instances are solely of children diagnosed with ASD. It, however, does include further annotations besides (1) vocalisations such as (2) speaking/non-speaking, (4) emotion, (3) ASD behaviour, and (5) non-verbal vocalisations. However, as we are only interested in ASD recognition from speech, we focus on the vocalisations and speech of the children. For the purpose of our experiments, we use all the child produced vocalisations, both speech and non-speech related. The total duration of the dataset samples utilised is 115 min.

### 2.2 Child pathological speech database (CPSD)

The child pathological speech database (CPSD) (24) was built with the goal of studying language-impaired children (LIC) diagnosed with ASD. It contains diagnoses of pervasive developmental disorder either of autism spectrum disorder (ASD), specific language-impairment such as dsphasia (DYS), or pervasive developmental disorder-not otherwise specified (PDD-NOS). Throughout this research we refer to (PDD-NOS) as (NOS). The recordings were collected from monolingual French-speaking children aged 6 to 18 years, in Paris, France (Université Pierre et Marie Curie/Pitié-Salpêtière Hospital and Université René Descartes/Necker Hospital). While a control group is introduced and it consists of 64 Typically Developing children (TD), 52 of which are males and 12 females. All the TD subjects had no prior history of difficulties with learning, speech, hearing or general learning. Recordings of all subjects were captured in their typical environment, i.e clinic for the LIC subjects and elementary, secondary, and high school for the TD subjects. The database was designed with the main aim of comparing and assessing the children’s language skills to replicate diverse types of intonations (descending, falling, floating and rising contours). This was achieved, examining intonation contours, by as- signing subjects with an imitation task which consisted of imitating 26 pre-recorded sentences representing different modalities (i.e., declarative, exclamatory, and interrogative).

To conduct our experiments on the typicality task, we combine vocalisations from children with Dysphasia specific language impairment (DYS), autism spectrum disorder (ASD) and pervasive developmental disorder-not otherwise specified (NOS) into one class (AD). As in this task we are merely interested in the automatic recognition of speech as of an AD or TD child. Additionally, the previous dataset has ASD as one class, therefore we combine instances collected of AD into a single class. For the diagnosis task, speech samples are classified into four distinct labels: TD, ASD, DYS, and NOS, treating each as a separate category. In total, the samples add up to 62 min of audio.

The distribution of the datasets over the training, development, and test sets for the typicality and diagnosis classification tasks is displayed in Table 1. We use a split of 70% training, 15% development, and 15% test, and further split the training set; 70% training, 15% female, and 15% male. The same split strategy is implemented to the four-class diagnosis classification task, therefore both tasks use the same folds, we do this to enable fair comparability between the two tasks. During the data pre-processing, we split the data according to speaker rather than samples, to ensure that samples from each child belong to one fold only (training, development, or testing sets). This step is initiated to prevent the model from over-fitting by learning speaker or recording idiosyncrasies, and therefore deceptively excelling. The De-Enigma corpus comprises significantly more vocalisation samples than the CPSD, however, lacks a control group. CPSD is 64%–36% AD-TD labels, this introduces a data imbalance, which must be addressed during pre-processing and network training.

**Table 1:** **Typicality:** Data distribution over different partitions and class categories of the DE-ENIGMA dataset, and CPSD.

Typicality					
DE-ENIGMA	∑	AD		TD	
Train	3 834	3 834			
Develop	793	793			
Test	741	741			
∑	5 368	5 368			
CPSD					
Train	920	223		697	
Develop	258	156		102	
Test	284	78		206	
Female	130	78		52	
Male	130	78		52	
∑	1 722	613		1 109	
**TOTAL**	**7 090**	**5 981**		**1 109**	
Diagnosis					
DE-ENIGMA	∑	ASD			
Train	3 834	3 834			
Develop	793	793			
Test	741	741			
∑	5 368	5 368			
CPSD	∑	DYS	ASD	NOS	TD
Train	920	51	104	42	723
Develop	258	78	26	52	102
Test	284	26	26	26	206
Female	130	26	26	26	52
Male	130	26	26	26	52
∑	1 722	233	208	172	1 109

AD stands for atypically developing; TD for typically developing. Note: The DE-ENIGMA TD column is blank, as the dataset does not include a control group. **Diagnosis:** Data distribution over different partitions and class categories of the DE-ENIGMA dataset, and CPSD. ASD stands for autism spectrum disorder, DYS for dysphasia, NOS for pervasive developmental disorder-not otherwise specified, and TD for typically developing.

Bold values indicates total refers to the sum across the two corpora.

The majority of the samples are of male samples, as opposed to female; this is expected, as there is an observed 4:1 male-to-female prevalence of ASD (25, 26). This could be due to the “female camouflage effect”, which suggests that females are better at masking ASD (27). The CPSD has 13 female participants making 334 samples and 54 males making 1,388 samples, creating a gender imbalance of 19%-81% in the data. The De-Enigma corpus similarly has a wide disparity with 11 females and 39 males, forming a 22%-78% percent split. Due to these gender disparities, we perform fairness testing on the proposed models.

## 3 Methods

### 3.1 Discriminative fine-tuning

Fine-tuning entails pre-training a source model on one task, then tuning it on a target model for another similar task. We implement fine-tuning to test whether we are able to obtain a good performing model without the need for extensive datasets and deep learning techniques. This is based on the assumption that, if two tasks are similar, some knowledge can be transferred between models. More specifically, a discriminative fine-tuning model is utilised, which is different from a generative adversarial network (GAN) (28) as it excludes a generative model. It is commonly used for classification and prediction tasks where explicit modelling of the data distribution is not required. We choose to implement a discriminative model as we are interested in task-specific performance; this bears the ability to fine-tune and optimise based on the unique characteristics of speech data. Furthermore, omitting the generative model enables the network to train faster and potentially perform better for the specific task at hand while focusing the discriminative model on learning the decision boundary between the classes.

**Figure 1:**
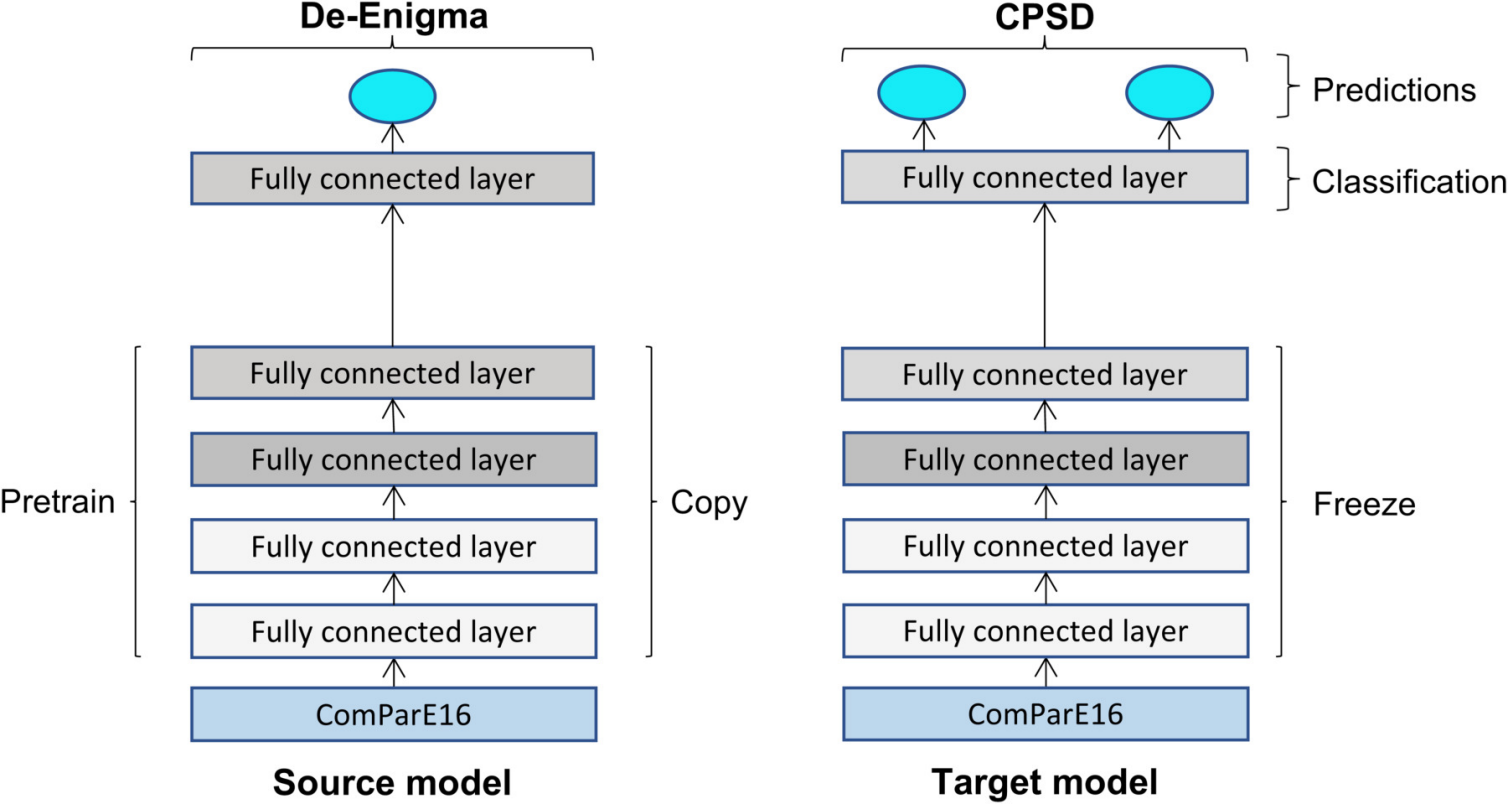
Schematic diagram of the fine-tuning adopted architecture for the source model pre-trained on the De-Enigma corpus and the target model fine-tuned on the CPSD corpus.

Figure 1 outlines the proposed architecture for training the discriminative fine-tuning model, which we refer to as the D-FT model. The network is pre-trained on the source model using the De-Enigma corpus, the same network parameters are shared by the target task, with a classification layer added with the number of classes of the target dataset. The new output layer in the target model is then trained on the CPSD data. The model is pre-trained using one class label, children diagnosed with ASD, then fine-tuned using two class labels, ASD vs. TD. The same model is utilised for the diagnosis task, but it is fine-tuned to classify speech into one of four categories: TD, ASD, DYS, or NOS, rather than using the binary TD vs. AD classification.

### 3.2 Wav2Vec 2.0 fine-tuning

Wav2Vec 2.0 introduced by Baevski et al. (19) is a self-supervised learning framework, where representations are learnt from raw audio data. It was first released as Wav2Vec for automatic speech recognition (15, 16). The pre-trained model can be fine-tuned to the specific task and dataset at hand. It is a transformer-based model that predicts audio samples based on quantisation. The model has the capability to outperform other models with an amount of transcribed speech (labelled data) as little as 10 min (29). We test this finding in our experiments, as the training set from the CPSD comprises of 30 min of labelled data.

**Figure 2:**
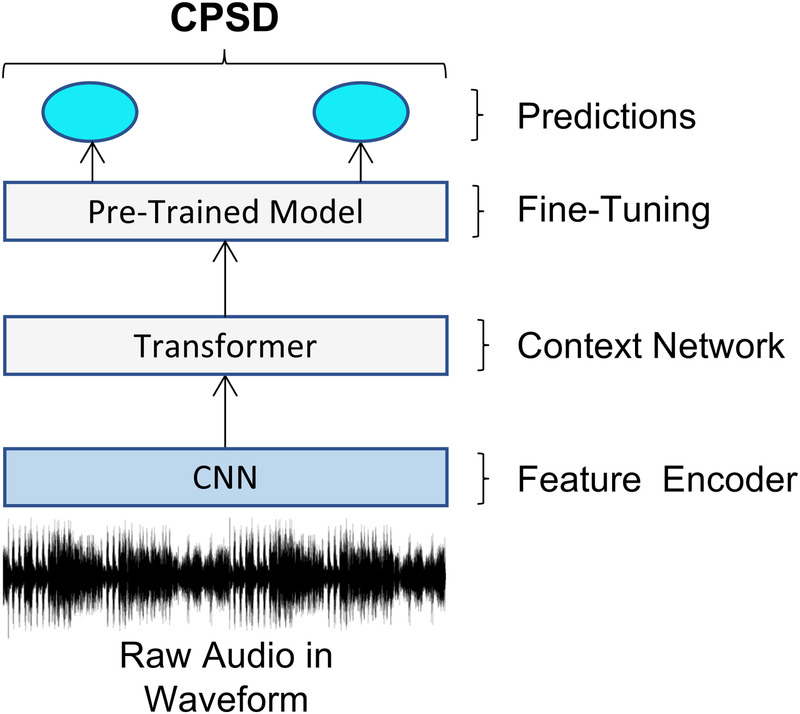
Schematic diagram of the adopted architecture for fine-tuning the Wav2Vec 2.0 model on the CPSD data.

Figure 2 outlines the framework for the Wav2Vec 2.0 fine-tuned model, which we refer to as the W2V2-FT model. Starting with raw waveform as input, we then produce latent space representations which are then fed to the transformer. We leverage the Wav2Vec 2.0 pre-trained model on the Libri-Speech corpus comprising of 960 h of speech (30) by using the **facebook/wav2vec2-large-librispeech-960h**, then fine-tune it using the CPSD data. The De-Enigma corpus is not used here as we are interested in the performance of a model pre-trained on a large, yet unrelated dataset, being Libri-Speech then fine-tuned to a small dataset, being CPSD, to use for comparisons to the previous model. The diagnosis task utilises the same model but is adapted for multi-class classification, distinguishing between TD, ASD, DYS, and NOS, to further test the model’s performance at multi-class classification.

## 4 Experimental setup

### 4.1 Discriminative fine-tuning

The audio is first pre-processed, from raw audio form to model comprehensive data. Acoustic feature extraction is preformed using ComParE16; a set comprising of 6,373 features created for the INTERSPEECH 2016 computational paralinguistics challenge (ComParE16) (31), available from the openSMILE (32) toolkit. These features capture various aspects such as spectral, energy, F0, and cepstral coefficients (MFCCs) and other frame-level features referred to as low-level descriptors (LLDs), enabling comprehensive analysis of aspects of speech. The speech features are then standardised, as this can enhance the model’s capability to generalise and avoid bias towards certain features. Before feeding the features to the model for training, we upsample due to the considerable class imbalances. For the typicality task, a factor of three is used to upsample the minority class (AD, encompassing all non-typically developing children). In the diagnosis task, a factor of five is applied to the minority classes (DYS, ASD, NOS), while the majority class (TD) remains unaltered.” The most suitable resampling technique for this model we find to be upsampling by repeating the sparse examples based on an upsampling dictionary. This is a useful technique, especially when dealing with sparse or low-resolution data. It is widely used in signal processing tasks, as it involves increasing the resolution of a signal or dataset by generating new samples.

### 4.2 Wav2Vec 2.0 fine-tuning

To employ a Wav2Vec 2.0 model, the audio samples need to be resampled to 16 kHz which is what the pre-trained model is trained on. The CPSD samples are already in 16 kHz form, therefore, we skip the resampling step. The model includes a CTCtokeniser for tokenising speech, which is then used by the CTCFeatureExtractor for feature extraction, it encompasses 1,024 features. Wav2Vec 2.0 utilises a convolutional neural network (CNN) as part of its architecture, more specifically for the component responsible for extracting features from the input audio data. Due to the class imbalances, the model is at risk of over-fitting by favouring the majority class over the minority. Therefore, we resample the data using various methods and examine the model’s behaviour in order to find the most suitable technique. These resampling methods include random (over and under) sampling, Synthetic minority over-sampling technique (SMOTE) (33), its variants support vector machine-SMOTE (SVM-SMOTE) and borderline-SMOTE (BL-SMOTE) (34), and finally adaptive synthetic (ADASYN) sampling (35). With random under-sampling, which is not always recommended due to important points in the dataset are being discarded, reducing sample variety and resulting in the model not being able to generalise to unseen data, the model struggles to avoid over-fitting to one label at the early epochs of training. Similarly, with the other techniques, the model over-fitted to the data fairly quickly, except for SVM-SMOTE for the binary typicality classification task, even though it does not produce an equal number of samples for the minority class to the majority class (see Table 2). The same network and parameters are used for the four-class diagnosis task, where ADASYN proved to be the most suitable resampling technique for handling class imbalances, primarily due to the significantly larger number of TD samples compared to ASD, DYS, and NOS. As displayed in Table 2, the majority class (TD) remains the same while other class are upsampled, though unequally.

**Table 2:** W2V2-FT training samples for the CPSD before and after employing resampling techniques; SVM-SMOTE for the binary typicality task and ADASYN for four-class diagnosis task.

Typicality					
SVM-SMOTE	∑	AD		TD	
Before	920	697		223	
After	1,244	697		506	
Diagnosis					
ADASYN	∑	DYS	ASD	NOS	TD
Before	920	77	104	42	697
After	2,630	693	726	714	697

## 5 Network training

### 5.1 Discriminative fine-tuning

We use a deep recurrent neural network (RNN) with gated recurrent units (GRU) with a discriminative loss utilising signmoid cross entropy with logits, and optimise with adam optimization (Adam optimizer) and a regularisation rate of 0.0001. Additionally, a dropout rate of 0.3 is introduced to the network. We use a grid search strategy to find the best performing hyperparameters, and although it is time-consuming, we found it to be more efficient and still requiring less time than manual random search. After testing various parameters, we use the following for our final model; five fully connected layers, a learning rate of 0.001, a batch size of 128, and 1,024 hidden nodes.

### 5.2 Wav2Vec 2.0 fine-tuning

The pre-trained model uses a dropout rate of 0.1, even though we test various rates and observe its behaviour, it performs best with 0.1. We use a learning rate of 0.0001, as we find that the model over-fits early in the training with larger rates. Once again, the Adam Optimization is applied, specifically adamw_torch. We further apply label smoothing, as it makes the model less sure of it predictions and thus aids the model in avoiding over-fitting. However, it did not necessarily improve this model’s performance, and we settle for no smoothing applied.

## 6 Results

Our experiments use two feature extraction methods, the ComParE16 feature set and the Wav2Vec2FeatureExtractor. We explore the performance of the feature extractors and the classifiers with their varying applied loss functions. To compare the results, as displayed in Table 3, the unweighted average recall (UAR) and F1-score of the models are considered. For the typicality classification task, the baseline achieves a UAR of 94.1%, which is indicative of the classification ability of deep learning models. For comparative reasons, the baseline utilises the same network and hyperparameters of the fine-tuning D-FT model using the CPSD data, as it includes the control group, excluding the pre-training step. We choose this baseline, as we further wish to explore the performance of deep learning models with relatively small sets.

**Table 3:** Results for F1 and UAR from the development and test sets. Typicality refers to the 2-class task (ASD vs. TD) and diagnosis refers to the 4-class task (DYS-ASD-NOS-TD).

[%]	Develop		Test	
Approaches	F1	UAR	F1	UAR
Typicality				
Baseline	73.8	77.2	93.1	94.1
D-FT	73.6	78.0	93.5	94.8
W2V2-FT	72.0	76.6	89.5	91.5
Diagnosis				
Baseline	33.7	36.3	56.1	62.4
D-FT	34.4	37.9	59.3	60.9
W2V2-FT	33.6	34.3	44.3	54.3

D-FT yields a 94.8% UAR score, which is the highest score from all approaches. Regardless of the De-Enigma corpus lacking a control group, the pre-trained model aids the classification done at the fine-tuning stage. The D-FT model being similar to the baseline in terms of utilising the same fine-tuning network is indicative of its ability to improve performance through the additional related source model (the auxiliary pre-training step), trained on the De-Enigma corpus. However, the improvement not being significant could mean that the model reached its potential with the given dataset. Whilst, the W2V2-FT model produces a UAR of 91.5%. The model was not as robust to over-fitting and generalisation when compared to the D-FT model. As discussed in Section 5.2, the model was prone to over-fitting at early epochs due to the class imbalance further proving that ML models are highly reliant on the quality and quantity of the datasets used for training. Furthermore, the feature extraction method used is also a factor in model performance. The ComParE16 feature set includes a higher number of features in comparison to the Wav2Vec2FeatureExtractor, which means that it can potentially capture more detailed and diverse information from the audio data.

For the diagnosis task, classification performance is significantly lower compared to the typicality task, likely due to the increased complexity of distinguishing between multiple conditions (ASD, DYS, NOS, TD) rather than a binary classification. The D-FT model performs less accurate predictions as the UAR score is 60.9%, which could be attributed to the additional dataset used. In the four-class diagnosis task, the De-Enigma corpus (which contains only ASD cases) does not fully align with the CPSD dataset, which includes a broader range of categories (DYS, ASD, NOS, and TD) Consequently, training multiple datasets that have inconsistencies in their categories hinders model performance (36). However, the baseline performs considerably better with 62.4% on the test UAR, even though it only uses the fine-tuning part of the D-FT model. Again, this could be due to the process of training the baseline not necessitating handling category mismatch. Finally, the W2V2-FT strategy yields the lowest scores in performance metrics, with a UAR of 54.3%. In this case, similar to the typicality task, the model benefits from the pre-trained model, regardless of the training being on unlabelled data and an unrelated task. On the other hand, the D-FT model could perform comparatively better due to the datasets utilised for training being of related tasks. We assume that the D-FT model is better suited to handling the additional complexity introduced by the four-class diagnosis task, whereas the other model may struggle to generalise across multiple conditions.

The diagnosis task, compared to the typicality task, is more complicated and not only because there are more classes. Even though the binary task suffers from class imbalance, the imbalance is more severe in the four-class task. Moreover, in a multi-class task there are more decision boundaries that the model needs to learn. However, in a binary-task the model is only required to learn one decision boundary to separate the two classes (AD and TD). Finally, the evaluation metrics in binary classification are more straightforward to interpret, compared to multi classification metrics for each class. As a result, the performance scores of the training models are lower than those of the binary class.

## 7 Discussion

Wav2Vec 2.0 has shown promising results when applied to speech classification, being Autism Spectrum Disorder recognition, although it was initially built for a different task, being automatic speech recognition. Upon analysing and contrasting the performances of our classification models, we observe that during the early training stages the D-FT model did not overfit to the data when the W2V2-DT model did, due to the class imbalances. Moreover, the discriminative model’s performance and behaviour was similar for both tasks even without resampling the data, for instance, similar accuracy was achieved in the typicality task. This can indicate that it is more robust than the Wav2Vec 2.0 model. After further comparisons, we conclude that training on a similar task using a dataset with a large number of samples transfers knowledge to the fine-tuned model training on a relatively smaller dataset. While knowledge is similarly transferred when fine-tuning the same dataset on a pre-trained model on a comparatively larger dataset of 960 h, it does not necessarily result in better performance compared to training two related tasks. Another notable difference in the datasets used for training the two networks is the labelling. Wav2Vec 2.0 is pre-trained on unlabelled data while our D-FT model benefits from the labelled De-Enigma data used at the pre-training stage. However, in the diagnosis task, the additional dataset used for pre-training the D-FT model did not improve predictive accuracy, likely due to differences in feature distributions among ASD, DYS, and NOS. Additionally, the datasets include three different settings, being Serbian, English, and French – the diversity can aid the models generalise to various settings and is more robust to language changes rather than singularity, such as English for instance.

In Table 4, a comparison to other works on CPSD is given. The CPSD dataset was used for the INTERSPEECH 2013 *Autism Sub-Challenge* (8); we therefore compare our baseline results to the challenge baseline and winners (37). For fair comparisons, we only compare our baseline model as it utilises the same dataset as the challenge without additional the training set, such as the other two models (D-FT and W2V2-FT). Comparisons between UAR for the test set are shown in Table 5. The challenge includes a four-class (DYS-ASD-NOS-TD) classification task using all dataset labels as well as two class (AD-vs.-TD) classification. The challenge baseline uses linear kernel support vector machines (SVM)/support vector regression (SVR) with sequential minimal optimization (SMO) for static classification (regression). The SVM complexity parameter C is selected from a set of values to optimise performance, and logistic models are fitted to SVM hyperplane distances for obtaining class posteriors. Resampling is employed to address the class imbalance, by upsampling the under-represented classes. In the diagnosis task, the minority classes (DYS, ASD, NOS) are upsampled by a factor of five, whereas in the typicality task, the AD class is upsampled by a factor of two to balance the dataset.

**Table 4:** Results compared to other research using the same dataset, being CPSD.

[%]	Test UAR	
Studies	Typicality	Diagnosis
Baseline	94.1	62.4
Schuller et al. (8)	90.7	67.1
Asgari et al. (37)	93.6	69.4

**Table 5:** Results of the W2V2-FT model compared to other research using an implementation of Wav2Vec2.

[%]				
Approaches	Accuracy	Precision	Recall	F1
W2V2-FT	91.2	88.0	91.5	89.5
Chi et al. (39)	76.9	78.2	74.6	76.8
Lee et al. (40)	71.7	73.1	60.5	66.2

In the study by Asgari et al. (37), a support vector classifier was used to recognise Autism Spectrum Disorder (ASD) cases, and support vector regression was employed to identify the subtypes. The regression and classifier models were trained using the open-source WEKA toolkit, with a hyperparameter value of C=0.001 retained from the baseline system. Similar to the challenge baseline, upsampling is implemented on the samples of the atypicality categories (DYS-ASD-NOS) by a factor of five. The feature extraction is done through: voice quality features (obtained through harmonic analysis), energy-related features, spectral features, and cepstral features. Our baseline model scores higher than the INTERSPEECH2013 autism sub-challenge research studies for the typicality task. Although our experiments and the challenge baseline use the ComParE feature set, in our experiments we employ a later version, released in 2016 while they use the 2013 set. This is indicative of the technological advancements in the AI field as one must remember that there is a decade gap between the experiments. As for the diagnosis task, neither does our base nor none of our models perform better classification than the other studies.

We compare our results, presented in Table 5, with the findings of other research studies that have employed different versions of the Wav2Vec 2.0 model for classifying ASD-vs.-TD, whereas our study focuses on AD-vs.-TD in the typicality task. While both approaches involve distinguishing between typical and atypical development, our classification includes a broader range of atypically developing children (ASD, DYS, NOS), whereas previous studies focus solely on ASD. An additional study by Hansen et al. (38) is excluded due to the lack of available scores, with reported F1-scores ranging between 0.54 and 0.75. Chi et al. (39) fine-tune the **facebook/wav2vec2-base** variant on crowd sourced semi-structured data, whereas we fine-tune the **facebook/wav2vec2-large-librispeech-960h** variant. While the study by Lee et al. (40) utilises the Wav2Vec 2.0 model for feature extraction and employs a bidirectional long short-term memory (BLSTM) classifier for the downstream task. Lee et al.’s W2V-BLSTM model has two versions: W2V-BLSTM-FT and W2V-BLSTM-JT. In W2V-BLSTM-FT, the BLSTM classifier is fine-tuned using the downstream task of ASD-vs-TD classification on the pre-trained base model on the LibriSpeech data. The quantisation process in the Wav2Vec 2.0 model is removed, and context representations are derived solely from the input signal. These context representations are extracted for a given waveform and used to train the BLSTM-based classifier. On the other hand, the W2V-BLSTM-JT model is trained by jointly optimising all parameters, including those of the Wav2Vec 2.0 model and the BLSTM-based classifier. This model combines the feature extraction capabilities of Wav2Vec 2.0 with the classification power of the BLSTM network. Our W2V2-FT model achieves comparatively higher UAR and accuracy scores than those reported in the studies reviewed in this work, underscoring the potential of our fine-tuning approach for distinguishing between typically and atypically developing children across a broader classification task. However, given the variation in datasets and methodologies across studies, direct comparisons should be interpreted with caution.

## 8 Fairness testing

Discrimination and bias are two of the main concerns regarding ethical AI, along with privacy and surveillance. With developing AI models, and more so algorithmic decision making, there is potential bias and discrimination that must be taken into account from design to generated predictions. This is crucial in our experiment, specially that we are adhering to a vulnerable group. The 4:1 male-to-female prevalence of ASD serves as an additional significant rationale for undertaking fairness testing. While this is expected behaviour of the model when it has more samples from one gender, tests must be performed to find ways to address and prevent the bias. Conclusively, in our data, there is a ratio of 8:2 male-to-female. This can bias the model analysis towards males as females are underrepresented in the data. For this reason, we perform fairness testing by testing equalised odds, which is where the protected and unprotected groups should have equal rates for true positives and false negatives (41, 42).

In order to test whether our model fairly classifies without bias, we analyse its performance in predicting AD among males-to-females. This was a challenging task as the female samples are significantly lower in comparison. This is carried out by analysing the model’s performance when tested on samples from one gender, noting that these samples must be new to the model and both models must be tested on the same female and male sets. The data distribution over the two genders is displayed in Table 1.

Testing for equalised odds is conducted from a confusion matrix by calculating and comparing true positive rates (sensitivity) to false positive rate (specificity) across the gender groups. Ideally, for model fairness, the disparity between the two groups should be relatively small. Sensitivity, also referred to as recall, is obtained by calculating the ratio of true positives (TP) to the sum of true positives and false negatives (TP+FN). Specificity is the ratio of true negatives (TN) to the sum of true negatives and false positives (TN+FP).

Mathematically, sensitivity is calculated as (see Equation 1):

(1) $$\text{Sensitivity}=\frac{TP}{TP+FN}$$

While specificity is calculated as follows (see Equation 2):

(2) $$\text{Specificity}=\frac{TN}{TN+FP}$$

**Figure 3:**
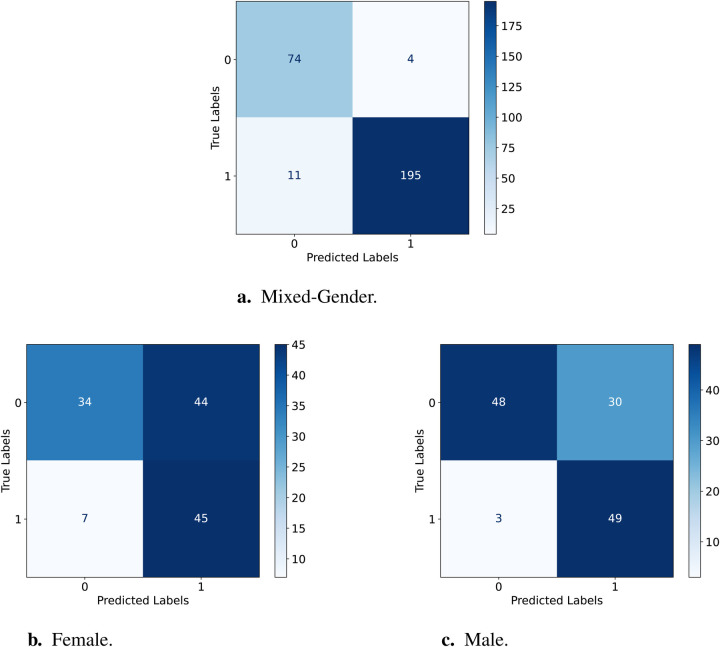
Confusion Matrices for testing the D-FT model on female vs. male. **(a)** Results for the female test set. **(b)** Results for the male test set. **(c)** Results for the mixed-gender test set.

**Figure 4:**
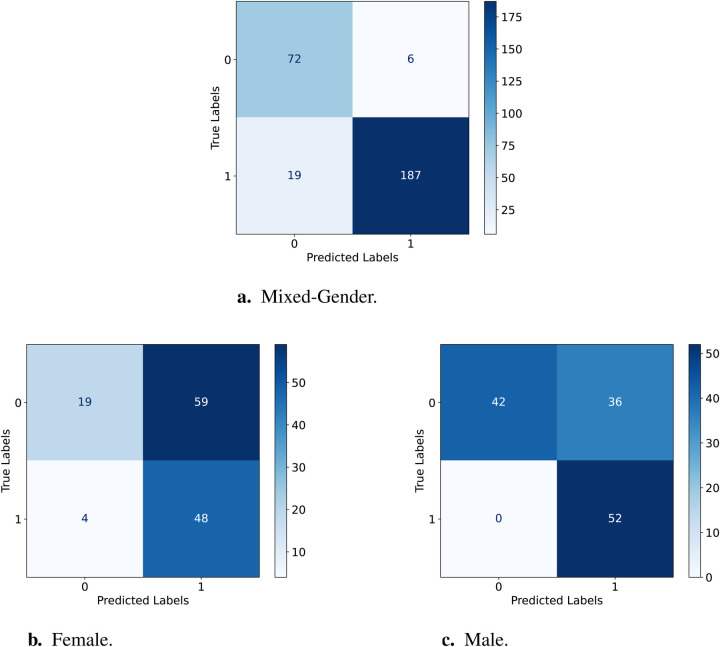
Confusion Matrices for testing the W2V2-FT model on female vs. male. **(a)** Results for the female test set. **(b)** Results for the male test set. **(c)** Results for the mixed-gender test set.

The confusion matrices for the testing sets from the CPSD dataset of female, male, and mixed-gender are used to compare the fairness performance of the D-FT and W2V2-FT models for the binary typicality classification task. The matrices with the true and predicted labels are displayed in Figures 3 and 4 which are used for the equalised odds testing.

Table 6 displays the sensitivity and specificity for D-FT and W2V2-FT across the two gender groups; for comparisons, we further include the ratios for the mixed-gender test set. Testing the D-FT model on the male set has a higher sensitivity than female, suggesting that the model performs better at correctly identifying AD for male compared to female. The same can be said for correctly identifying TD, this is indicated by the higher specificity for male compared to female. As for the W2V2-FT model, sensitivity and specificity are also lower for females and higher for males. This shows that the W2V2-FT model is less effective in correctly identifying AD and TD for females, but better for males. Comparatively, the W2V2-FT model displays lower sensitivity scores than those of the D-FT model, suggesting its reduced capacity at precisely recognising AD within both genders. Finally, the specificity for the W2V2-FT model being higher for both genders than the D-FT model suggests that W2V2-FT better captures AD instances for each gender. However, it is evident that both models perform better identifying TD than ASD.

**Table 6:** **Typicality:** Sensitivity and specificity percentages for equalised odds fairness testing across models and gender groups.

Typicality		
Model	Sensitivity	Specificity
D-FT		
Mixed-Gender	94.8	94.7
Female	43.6	86.5
Male	61.5	94.2
W2V2-FT		
Mixed-Gender	92.3	90.8
Female	24.4	92.3
Male	53.8	100

The testing results, displayed in Table 7, indicate that the models performed considerably better for males than females, revealing a potential gender bias in classification accuracy. This suggests that the imbalance in training data, where male samples are overrepresented, may have influenced model generalisation. The uneven gender distribution in the dataset likely impacted the model’s ability to generalise across both genders, giving the majority group (males) an advantage over the minority group (females) and resulting in better predictions. The testing metrics further indicate that the D-FT model outperforms the W2V2-FT model in all instances, except for male testing in the diagnosis task.

**Table 7:** Performance results of the D-FT and W2V2-FT models on the female and male test sets.

Typicality				
[%]				
Test Set	Accuracy	Precision	Recall	F1
D-FT				
Mixed-Gender	94.7	79.5	94.8	93.5
Female	60.8	66.7	65.1	60.5
Male	74.6	78.1	77.9	74.6
W2V2-FT				
Mixed-Gender	91.2	88.0	91.5	89.5
Female	51.5	63.7	58.3	40.9
Male	72.3	79.5	76.9	72.1
Diagnosis				
[%]				
Test Set	Accuracy	Precision	Recall	F1
D-FT				
Mixed-Gender	81.7	62.0	60.9	59.3
Female	43.8	42.2	33.7	32.8
Male	50.8	40.9	41.3	38.9
W2V2-FT				
Mixed-Gender	64.8	43.3	54.3	44.3
Female	28.5	30.2	21.2	23.4
Male	65.4	63.3	58.7	54.6

Conducting fairness testing on the models for the diagnosis task displays similar behaviours to the typicality task. For instance, all the training models perform better classification for males than females. We further observe that in the diagnosis task, testing only male samples on the W2V2-FT model yields marginally higher scores compared to testing a combined-gender dataset. This implies that the female samples in the mixed-gender test set hinders performance, noting that the mixed set consists of more samples. In fact, the model struggles to correctly identify AD cases for females, also expected with the gender sparsity in the dataset.

Previous work predicts that there exists a subtle yet discernible gender bias with end2end models such as Wav2Vec2 (43). Our work supports this, as the W2V2-FT model was unable to perform similarly for both genders due to the unbalance and considerably larger portion of the data being of males. The same applies to both models in this study. Additionally, from the confusion matrices above we can deduct that both models are better at predicting TD than AD, presumably due to the dataset comprising of more TD samples. This is evident from the models in Figures 3 and 4 not predicting AD as TD.

## 9 Limitations

The initial aim of the study was autism recognition from earlier ages between 1 and 3 years; however, as aforementioned in Section 1, obtaining datasets with sensitive subjects is difficult and thus the course of research was changed to older ages between 6–18 years. However, pre-linguistic vocalisations have linguistic value, and the same applies to individuals on the spectrum as non-verbal vocalisations are a form of speech and hold communicative value (44). Moreover, analysing infant vocalisations can facilitate early ASD detection (45), which can be as early as the pre-linguistic vocalisation they produce (46). The D-FT model utilises both speech and non-speech vocalisations from the De-Enigma corpus, suggesting its potential to predict vocalisation patterns in children younger than four years old.

One of the limitations of this study is that the data in the two datasets is collected at different settings. The De-Enigma corpus was collected at-home, while the CPSD was recorded in lab-settings. Speech collected from a child’s natural environment, however, such as home, is more accurately reflective of their real life experience, which explains the massive shift we have been witnessing in recent years towards collecting and analysing day-long audio recordings (47). On the other hand, difference in data collection settings can help in generalising to various environments, while also potentially leading to a domain shift, meaning that the characteristics of the data may vary significantly between the two settings. Models trained on diverse datasets may struggle to generalise well to unseen data, as they might not effectively capture the variations and characteristics specific to each setting. As the D-FT uses both dataset, it is at risk of domain discrepancy which can result in reduced performance when the model encounters data from either setting. Additionally, as one dataset has significantly more samples, the D-FT model, which utilises both datasets, could potentially become more biased towards the more dominant setting.

Class imbalances in the CPSD dataset was limiting the performance of the W2V2-FT model, with a 64% of the samples being TD and 34% being of AD. Although we address this in the pre-processing and network training with methods such as resampling, the W2V2-FT model performance deteriorates. Resampling methods including SMOTE and ADASYN can be beneficial in some tasks, while also insufficient in other applications (48). Moreover, methods such as SMOTE affect classification performance (49). The same can be said regarding the data used for the diagnosis task, class imbalances did hinder prediction accuracy. There is a further imbalance in the gender distribution in our datasets, having substantially more male participants, with 95% in the De-Enigma corpus and 90% in the CPSD. To investigate this, we perform fairness testing, resulting in the models performing better classification within male than female as they are trained on more male samples. Additionally, the experiments evaluate and test on relatively small sets, which could hinder the model improvement and possibly meaning it is not fully representative of the real-world.

The UAR results of 78.0% on the development set and 94.8% on the test set, for the D-FT model for instance, while can be considered quite promising, they should be considered in the context of the dataset’s specific characteristics. The CPSD is relatively small, and we have additional splits (i.e., female and male sets), which can lead to exhibiting more substantial variation in performance. Additionally, we are dealing with a class imbalance, where TD has more samples than AD. This class imbalance can make it more challenging for the model to perform predictions, especially for the minority class. In the training set this imbalance was addressed by upsampling the minority class, to enable learning better representation of this class. Whilst the development and test sets remain intact with their distributions unchanged, to be representative of real-world. By initiating this, the evaluation of the model’s performance reflects on the actual class distribution encountered in the real-world application. However, due to the limitations in the dataset, the results reported herein must be carefully interpreted. For future work, stratified sampling can be utilised to mitigate the imbalance in the various folds. Stratified sampling is a method of sampling, it involves dividing a population into subgroups (strata) based on certain characteristics (class in our case) and then selecting samples from each group (stratum) to ensure representation of the entire population.

Most importantly, however, the accuracies reported herein are partially very high and have to be interpreted very carefully – they depend on various factors and the specific test data set and cannot easily be generalised to real-world diagnosis of autism condition, which can be expected to significantly lower accuracies – see also (50) for a discussion of potential over-expectation in speech analysis tasks for real-world application.

## 10 Conclusions

The importance of early detection and speech in ASD pose the point of our research. We therefore tested models based on fine-tuning algorithms for classifying children with AD and TD from their vocalisations and speech. The performance of the models indicates the computational abilities of modern-day deep learning models in the recognition of ASD from speech. We found that deep transfer learning through fine-tuning helped in leveraging multiple datasets and thus aided model performance in every instance for the typicality task. However, differences in class distributions between datasets in the diagnosis task negatively impacted predictive accuracy, particularly due to the challenge of integrating a dataset containing only ASD cases (De-Enigma corpus) with a dataset that includes a broader range of atypical developmental conditions (CPSD: ASD, DYS, and NOS). We further observed that performance was impacted by the data used in training and that resampling techniques, in the case of addressing class imbalances, were highly influential on the performance. Finally, testing the fairness of a model is crucial, especially when sensitive groups are involved but underrepresented in the data used for developing a model. The fairness testing further proved that models can be reliant on the data, as with more male samples in the datasets the models were biased towards males.

Future work could explore multi-modal classification by incorporating additional modalities such as facial expressions, as this study focuses solely on speech-based uni-modal classification. The De-Enigma dataset comprises further features which could be leveraged; facial mapping coordinates, speech and vocal noises, body posture as well as angle and rotation of the child’s head. Adding further classification features could possibly improve the model’s classification and prediction. Moreover, the class and gender imbalances in the datasets can be tackled by collecting more data. This will confront the over-fitting problem and better capture the diversity and characteristics present in tangible environments. Another plausible solution to the class imbalance is to utilise GANs to generate synthetic data samples. When compared to SMOTE and ADASYN resampling techniques, GANs have been found to be more representative of real-world samples (51).

## Data Availability

The original contributions presented in the study are included in the article/Supplementary material, further inquiries can be directed to the corresponding author/s.
